# A Novel Biosensing Approach: Improving SnS_2_ FET Sensitivity with a Tailored Supporter Molecule and Custom Substrate

**DOI:** 10.1002/advs.202303654

**Published:** 2023-10-20

**Authors:** Sobia Nisar, Beriham Basha, Ghulam Dastgeer, Zafar M. Shahzad, Honggyun Kim, Iqra Rabani, Aamir Rasheed, M. S. Al‐Buriahi, Ahmad Irfan, Jonghwa Eom, Deok‐kee Kim

**Affiliations:** ^1^ Department of Electrical Engineering Sejong University Seoul 05006 Republic of Korea; ^2^ Department of Convergence Engineering for Intelligent Drone Sejong University Seoul 05006 Republic of Korea; ^3^ Department of Physics College of Sciences Princess Nourah bint Abdulrahman University P. O Box 84428 Riyadh 11671 Saudi Arabia; ^4^ Department of Physics and Astronomy Sejong University Seoul 05006 Republic of Korea; ^5^ SKKU Advanced Institute of Nanotechnology (SAINT) and Department of Chemical and Polymer Engineering Sungkyunkwan University Suwon 16419 Republic of Korea; ^6^ Department of Chemical and Polymer Engineering University of Engineering & Technology Faisalabad Campus Lahore 38000 Pakistan; ^7^ Department of Semiconductor Systems Engineering Sejong University Seoul 05006 Republic of Korea; ^8^ Department of Nanotechnology and Advanced Materials Engineering Sejong University Seoul 05006 Republic of Korea; ^9^ School of Materials Science and Engineering Anhui University Hefei Anhui 230601 People's Republic of China; ^10^ Department of Physics Sakarya University Sakarya 54187 Turke; ^11^ Department of Chemistry College of Science King Khalid University P.O. Box 9004 Abha 61413 Saudi Arabia

**Keywords:** 2D semiconductors, biosensors, field‐effect transistors, hexagonal boron nitride, tin disulfide

## Abstract

The exclusive features of two‐dimensional (2D) semiconductors, such as high surface‐to‐volume ratios, tunable electronic properties, and biocompatibility, provide promising opportunities for developing highly sensitive biosensors. However, developing practical biosensors that can promptly detect low concentrations of target analytes remains a challenging task. Here, a field‐effect‐transistor comprising *n*‐type transition metal dichalcogenide tin disulfide (SnS_2_) is developed over the hexagonal boron nitride (h‐BN) for the detection of streptavidin protein (Strep.) as a target analyte. A self‐designed receptor based on the pyrene‐lysine conjugated with biotin (PLCB) is utilized to maintain the sensitivity of the SnS_2_/h‐BN FET because of the π–π stacking. The detection capabilities of SnS_2_/h‐BN FET are investigated using both Raman spectroscopy and electrical characterizations. The real‐time electrical measurements exhibit that the SnS_2_/h‐BN FET is capable of detecting streptavidin at a remarkably low concentration of 0.5 pm, within 13.2 s. Additionally, the selectivity of the device is investigated by measuring its response against a Cow‐like serum egg white protein (BSA), having a comparative molecular weight to that of the streptavidin. These results indicate a high sensitivity and rapid response of SnS_2_/h‐BN biosensor against the selective proteins, which can have significant implications in several fields including point‐of‐care diagnostics, drug discovery, and environmental monitoring.

## Graphical abstract

This study introduces a biosensing device composed of Tin sulfide (SnS_2_) and h‐BN crystals for streptavidin detection. Researchers incorporate a biotin‐based supporter construct conjugated with Pyrene–Lysine, effectively capturing the target analyte. Device performance is validated by Raman spectroscopy and electrical characterizations, which detect 0.5 pm streptavidin in 13.2 s.

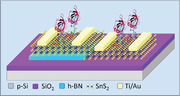

## 1 Introduction

The world is glutted with highly contagious virulent diseases that are a persistent hazard to human health and societal interactions around the globe, with some of these diseases having the potential to be lethal. For instance, the severe acute respiratory syndrome coronavirus 2 (SARS‐CoV‐2) caused coronavirus disease (COVID‐19) began to pose a high risk to the public's health globally in late 2019.^[^ ^1^ ^]^ Due to the lack of suitable treatment and testing systems, this virus spread rapidly across most continents and was subsequently recognized as a pandemic in March 2020.^[^ ^2^ ^]^ Moreover, serious brain diseases including Parkinson's and Alzheimer's are two serious brain disorders brought on by irregular alterations in the level of dopamine (3,4‐dihydroxy phenethylamine). These two most common neurogenerative diseases are not curable and lack neuroprotective detection and treatments. Their estimated worldwide frequency is 1% to 2% right now and is expected to increase over the coming decades.^[^ ^3^, ^4^ ^]^ Such pandemic diseases carry a heavy personal, societal, and financial impact on human life.^[^ ^5^, ^6^ ^]^ Therefore, for bringing a better health monitoring and remedial structure, the development of pertinent research tools and point‐of‐care devices (POC) is highly important, they can provide an ideal platform with an economical, reliable, and user‐friendly interface for the early screening of pandemics and other health concerns.^[^ ^7^, ^8^, ^9^, ^10^ ^]^ Conventional methods for protein‐based molecular diagnostic processes, such as immunoassays are expensive and labor‐intensive.^[^ ^10^, ^11^, ^12^ ^]^ Even though highly professional workers and meticulous sample preparation are essential to use these approaches.^[^ ^13^ ^]^ Because of these limitations, their use as a POC‐based system has been constrained and is now partially functional for providing service in a pandemic crisis.^[^ ^14^ ^]^ Subsequently, efficient biosensors that can promptly identify and characterize the pathogens using a small amount of target analytes are essential in a continuously growing epidemiology.^[^ ^15^ ^]^

Various biosensors have been extensively utilized in a variety of applications including food analysis,^[^ ^16^ ^]^ cancer diagnosis,^[^ ^17^ ^]^ toxin detection,^[^ ^18^ ^]^ and health prognosis.^[^ ^19^ ^]^ Field‐effect transistors (FET) are an appealing selection for devising fast, sensitive, and selective biosensors.^[^ ^20^ ^]^ For FET‐based biosensors, just a few test targets are needed^[^ ^21^ ^]^ for clinical diagnosis, point‐of‐care testing, and on‐site detection.^[^ ^22^, ^23^ ^]^ For instance, a recently developed graphene‐based FET biosensor with a reported limit of detection (LOD) up to 1 fgmL^−1^ has been presented as a tool for the prompt detection of SARS‐CoV‐2‐related spike protein.^[^ ^1^ ^]^ However, at times, graphene‐based biosensors provide erroneous indications due to the off‐state current leakage because of its zero bandgap.^[^ ^24^, ^25^ ^]^ Therefore, in addition to graphene, another resource that is recognized as an attractive material for biosensing is the 2D‐TMDCs (2D‐transition metal dichalcogenides) semiconductor.^[^ ^26^ ^]^ Contingent on the 2D materials, TMDCs have shown considerably tunable bandgaps ranging from milli‐electron volts (meV) to a few electron volts (eV), capable of lowering off‐state current while improving signal‐to‐noise (S/N) ratios.^[^ ^27^, ^28^, ^29^, ^30^, ^31^, ^32^ ^]^ Moreover, due to 2D‐TMDC's favorable optical, mechanical, and electrical characteristics, they have applications in various fields such as electronics, optoelectronics, energy storage, and biosensing.^[^ ^33^, ^34^, ^35^, ^36^ ^]^ Among the family of TMDCs, tungsten diselenide (WSe_2_) and molybdenum disulfide (MoS_2_) based FET has been utilized for precise and prompt analyte detection recently.^[^ ^10^, ^37^ ^]^ Additionally, MoTe_2_‐based FET exhibits good performance against selective protein detection^[^ ^36^ ^]^ and is also used for other applications like gas sensing^[^ ^38^ ^]^ and photodetection.^[^ ^39^ ^]^ TDMC materials are considered promising for biosensing applications due to their properties like large surface area and the possibility of direct interactions with various molecules.^[^ ^40^ ^]^ Moreover, TMDCs have been demonstrated to exhibit high surface‐enhanced Raman scattering (SERS) activity, which has drawn interest in the development of TMDC‐based biosensors.

In this research, we developed a field‐effect transistor composed of *n*‐type transition metal dichalcogenide (TMDC) tin disulfide (SnS_2_) over the hexagonal boron nitride (h‐BN) for the selective detection of the streptavidin protein (Strep.). The detection capabilities of the SnS_2_/h‐BN FET biosensors are investigated using both Raman spectroscopy and electrical characterizations. The self‐designed PLCB receptor binds to the SnS_2_ surface via π–π stacking, eliminates the screening effects, and preserves the sensitivity of the SnS_2_/h‐BN FET biosensor to detect the target analyte. The real‐time electrical measurements exhibit that the SnS_2_/h‐BN FET is capable of detecting streptavidin at a remarkably low concentration of 0.5 pm, within 13.2 s. Additionally, the selectivity of the device is investigated by measuring its response against a Cow‐like serum egg white protein (BSA) that is selected due to its near identical molecular weight with the streptavidin and Lysozyme (a mucosal secretion protein with different molecular weight). The high sensitivity and rapid response of the SnS_2_/h‐BN FET biosensor against the selective proteins has potential application in various fields such as point‐of‐care diagnostics, and epidemics.

## 2 Results and Discussion

### 2.1 Device Fabrication and PLCB Synthesis

**Figure 1:**
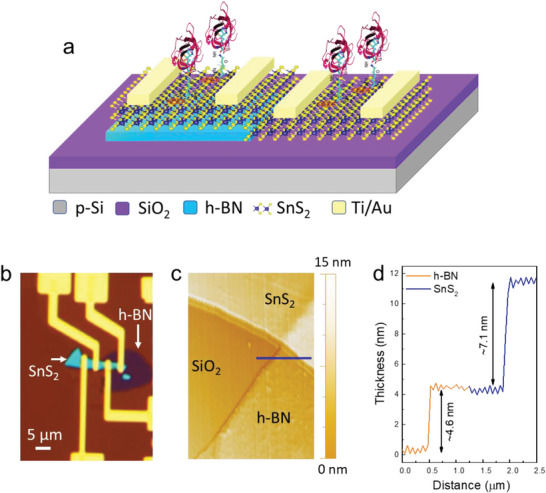
a) A schematic demonstration of the SnS_2_ FET device over h‐BN and SiO_2_ substrates for detecting selective protein (SA). Once the device had been directionally functionalized using our specially formulated PLCB supporter molecule, a solution containing streptavidin as a target analyte was deposited onto the device for detection. b) Optical image of the final device containing SnS_2_ over both substrates of h‐BN and SiO_2_ (scale bar represents 5 µm). c) The atomic force microscopic image (AFM) for the analysis of morphology and material thickness of h‐BN and SnS_2_. d) AFM height profiles revealed that the thickness of h‐BN was ≈4.6 nm, while that of SnS_2_ was ≈7.1 nm.

To prepare the devices, a mechanical exfoliation technique was used to peel off thin sheets of the 2D materials, allowing the production of ultra‐thin sheets up to a few layers thick. The SnS_2_/h‐BN FET devices are fabricated on a *p*‐doped Si/SiO_2_ substrate by transferring a few‐layer‐thick h‐BN followed by SnS_2_ onto it. Patterning of the electrodes is achieved using both photolithography and electron beam lithography techniques, with Ti/Au metal electrodes deposited on the SnS_2_ flakes using thermal evaporators in a high‐vacuum chamber for the desired metal deposition. The Ti layer serves as an adhesion layer and also reduces the work function, while the thicker Au layer provides good electrical conductivity.^[^ ^41^ ^]^ **Figure** **1**a,b depicts the final device's optical image and schematic diagram.

**Figure 2:**
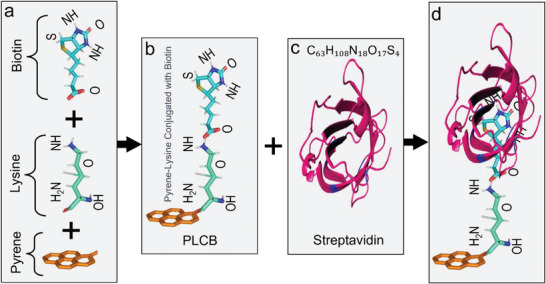
A visual representation depicting the a) structure and chemical formula of Pyrene, Lysine, and Biotin separately. Pyrene consists of four benzene rings, Lysine is an amino acid that contains a positively charged amino group and a carboxyl group. It has a flexible aliphatic side chain that contains a primary amine group, and Biotin is a vitamin that contains a heterocyclic ring fused to a tetrahydrothiophene ring. It has a valeric acid substituent on one end of the molecule, which can be used for conjugation with other molecules. b) Pyrene–Lysine conjugated with Biotin. c) Streptavidin protein with its chemical formula. d) Final schematic diagram of the conjugation of the PLCB supporter constructs after capturing the streptavidin protein.

The synthesis procedure and the structural code of our engineered PLCB supporter construct are presented in Figure S1a–c (Supporting Information). The PLCB receptor is a combination of the Pyeren‐lysine conjugated biotin as shown in **Figure** **2**a,b, in which a hexagonal pyrene ring attached at the bottom is utilized for the π–π stacking over the surface of hexagonal SnS_2_ materials. The PLCB molecule has a high electron affinity attributed to the presence of functional groups with electron‐withdrawing properties, such as the carboxylate and phosphate groups in the biotin part of the molecule. These functional groups have a strong ability to pull electrons away from neighboring atoms or molecules, which can make the molecule more electron‐rich and lead to a stronger attraction to additional electrons. The high electron affinity of the PLCB molecule could be relevant to its interactions with other materials, as it may tend to donate or accept electrons depending on the specific environment and conditions. The PLCB receptor is designed in such a way that it has the highest tendency to capture streptavidin through specific binding interactions between the biotin group of the receptor and the streptavidin protein as illustrated in Figure 2c,d. As streptavidin is a tetrameric protein that binds with high affinity to biotin (details are presented in Figure S2, Supporting Information). The biotin‐binding site is located in a pocket within the protein, and it is known to be very stable and specific. The biotin attached at the top of the pyrene–lysine assembly takes advantage of this high‐affinity interaction to capture the streptavidin.^[^ ^29^, ^42^ ^]^

### 2.2 Material Characterization

We examined the sample's chemical and physical properties with Raman spectroscopy and atomic force microscopy (AFM). To analyze the topography and thickness of each 2D material, atomic force microscopy (AFM) is a powerful tool that provides information at the nanoscale level. The AFM analysis of both SnS_2_ and h‐BN materials is presented in Figure 1c,d, which reveals their smooth surface and thickness. The thickness of SnS_2_ and h‐BN flakes was estimated to be ≈7.1 and 4.5 nm, respectively. based on the height profile graphs obtained from AFM analysis.

**Figure 3:**
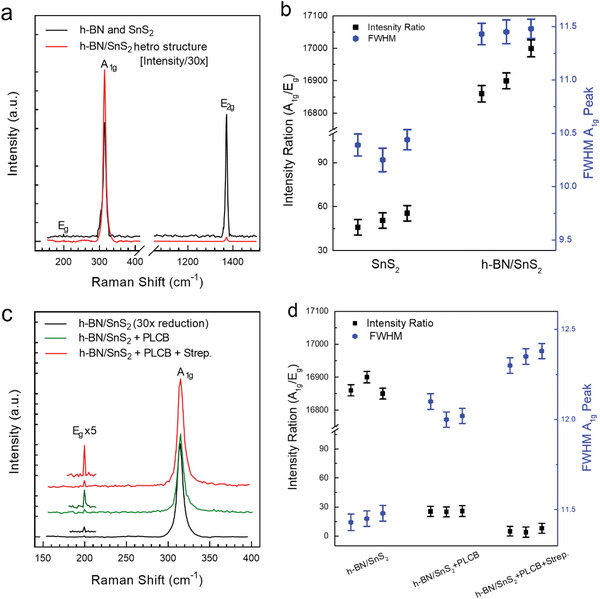
Raman spectroscopy analysis of the SnS_2_ and h‐BN biosensor device. a) The spectra of pristine SnS_2_ & h‐BN are presented in BLACK and are superimposed by the Raman spectra of the substrate containing SnS_2_ onto h‐BN as a substrate, presented in RED. b) For comparison, the intensity of SnS_2_ peaks has been increased by 30 folds when it is stacked over the h‐BN substrate (presented on the left in black). The FWHM for the *A*_1g_ peak is also increased slightly (presented on the right in blue color). c) The Raman spectrum of the pristine SnS_2_ flake over the h‐BN substrate (black line), while the green and red lines show the Raman spectra after functionalization and after streptavidin detection, respectively. The *E*_g_ peak is five times magnified to present the variation before and after streptavidin detection. d) The *A*_1g_/*E*_g_ peaks intensity ratio and FWHM are plotted in black and blue colors, respectively. The peaks were analyzed with Gaussian fit using Origin Pro 9.0 software, with sigma1 values (σ)> 6 and coefficient of determination (R^2^)> 95. The data was measured at three different devices (n = 3) to test consistency, as shown in Figure 3b,d, with standard error of the mean (SEM) ≤0.09, standard deviation (SD) ≤0.17, variance (P) ≤0.029, and coefficient of variance (CV) ≤9.80. A reliable and significant measurement has SEM near zero, SD ± 2, P ≤0.01, and CV <30, statistically.

Raman spectroscopy is a powerful analytical technique that is widely utilized due to its ability to provide useful information about the quality and surface analysis of materials, as well as its non‐destructive nature and sensitivity.^[^ ^43^, ^44^ ^]^ Therefore, the Raman spectra for the SnS_2_ and h‐BN are recorded at room temperature using a 532 nm laser and a 50x (and 100x) objective lens. The Raman spectra are measured at each stage: the device with pristine SnS_2_, h‐BN, the device after functionalization with our pyrene‐based receptor PLCB, and the device after capturing the target biomolecule (streptavidin). The detailed Ramans analysis for the h‐BN and SnS_2_ over the SiO_2_ substrate is illustrated in Figure S3a–d (Supporting Information). The spectra of pristine SnS_2_ & h‐BN are presented in **Figure** **3**a and are superimposed by the Raman spectra of the substrate containing SnS_2_ onto h‐BN as a substrate. The results revealed that the intensity of the SnS_2_ peak has been increased by 30 folds after stacking over the few layers of thick h‐BN. It can be observed that the peak intensity ratio of SnS_2_ (*A*_1g_/*E*_g_) has increased significantly from 45 to 17 000, i.e., ≈400% with an increase in full‐width half maxima (FWHM) as shown in Figure 3b. This significant change can be due to the increase in the sensitivity of the device^[^ ^45^ ^]^ by using h‐BN as a substrate for SnS_2_. Moreover, the high peak intensity of SnS_2_ over the h‐BN substrate may be attributed to the fact that h‐BN can bind stiffly with the SnS_2_ due to same hexagonal structure that leads to a better interaction and a sharp interface resulting in the increase in the ability of composite to resist micro‐deformations and dangling bonds.^[^ ^46^, ^47^, ^48^, ^49^, ^50^ ^]^ This combination will lead to less strain‐induced broadening of the Raman peaks and potentially higher peak intensity. Additionally, the lattice mismatch between SnS_2_ and h‐BN is lower as compared to SiO_2_, which can also affect the peak intensity.^[^ ^45^, ^51^, ^52^ ^]^ However, the maximum (FWHM) of the main resonance peak (*A*_1g_) has been increased insignificantly, depicting a slight loss in crystallinity of SnS_2_ material. The variation of peak intensity ratio and FWHM has been noted at the number of various positions and has been presented as an average of 3 data points showing consistency in measurement. On the other hand, the Raman spectra and peak properties of the *E*_2g_ peak (corresponding to h‐BN) have not changed significantly.

The spectra of pristine SnS_2_ over h‐BN are presented in BLACK and are superimposed by the Raman spectra of the substrate after its functionalization using our PLCB construct (GREEN) and after target protein detection (RED) as shown in Figure 3c. The properties of sharp peak (*A*_1g_) at ≈315.4 cm^−1^ were compared at different stages of device operation. The crystallinity of (*A*_1g_) is compromised, indicated by an increase in its FWHM, i.e., from 11.4 to 12.01 upon functionalization and up to 12.30 upon protein detection. However, the increase in this FWHM is not significant. Meanwhile, the peak intensity ratio (*A*_1g_/*E*_g_) has declined significantly^[^ ^29^, ^36^ ^]^ due to the attachment of our pyrene‐based supporter construct as presented in Figure 3d.

### 2.3 Gate Modulated Electrical Transport

**Figure 4:**
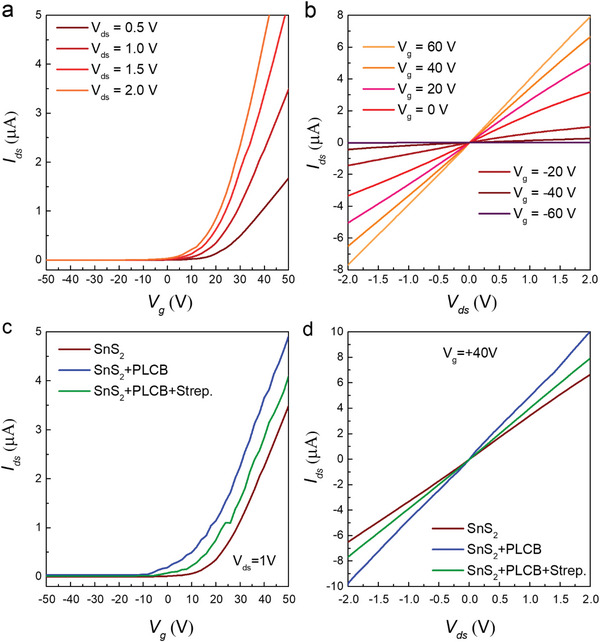
Electrical characterizations of the SnS_2_ FET over h‐BN substrate. a) The transfer curve of the pristine SnS_2_ FET over h‐BN at various *V*_ds_ (ranging from 0.5 to 2 V) and gate voltage sweeps spanning from −50 to +50. b) The output characteristics (*I*_ds_–*V*_ds_ curve) of the pristine h‐BN‐SnS_2_ device at various gate voltages (+60 to −60 V). The linear output curves show the Ohmic contribution of the Ti/Au metal electrodes with the SnS_2_ flakes. c) The comparison of the transfer curves of pristine SnS_2_/h‐BN (in brown color), SnS_2_/h‐BN functionalized with the PLCB supporter construct (in blue color), and SnS_2_/h‐BN with PLCB and streptavidin (in green color) at a fixed *V*_ds_ = 1 V. d) *I*_ds_–*V*_ds_ curves at a fixed gate voltage of *V*_g_ = +40 V of the pristine SnS_2_/h‐BN (in brown color), after functionalizing with the PLCB supporter construct (in blue color) and after introducing the streptavidin as a biomolecule (in green color).

The electrical characterizations of the final SnS_2_ devices are performed over SiO_2_ and h‐BN substrates, utilizing the Keysight B1500‐A semiconductor parameter analyzer and a probe station. To estimate the output current flowing through the SnS_2_ FET device, a biasing voltage (*V*_ds_) is applied to the source electrode, and drain current (*I*_ds_) is recorded through the drain electrodes. The gate voltage (*V*_g_) is applied to the substrate through its bottom via the *p*‐Si to produce an effective electric field that modulates the fermi level and charge transport through the channel material. The electrical characterizations of the pristine SnS_2_ over the Si/SiO_2_ substrate are illustrated in Figure S4a–c (Supporting Information). The transfer curves for a pristine device (SnS_2_ over h‐BN/SiO_2_) are recorded and illustrated in **Figure** **4**a, sweeping the gate voltages from −50 to +50 at various biasing voltages (*V*_ds_ = 0.5, 1.0, 1.5, and 2.0 V). The SnS_2_ FET shows a threshold in current at the positive gate voltage. Therefore, the transfer curves demonstrate a pure *n*‐type behavior of the device, which is attributed to the electrons as the majority of charge carriers. The carrier density of electrons (n) for pristine SnS_2_ is estimated by using the following Equation 1:^[^ ^53^, ^54^, ^55^ ^]^

(1) $$n=q^{-1}\;C_{g}\;\left|V_{g}-V_{\mathrm{th}}\right|$$

here, n represents the charge carrier density, which refers to the number of electrons per unit volume that can move freely through the SnS_2_ channel material, q is the elementary charge on an electron, *C*_g_ = gate capacitance, and *V*_th_ = threshold voltage, i.e., the minimum gate voltage needed to induce a significant change in the charge carrier density. A high charge carrier density (n = 6.25 × 10^12^ cm^−2^) was estimated over the h‐BN substrate.

Furthermore, as illustrated in Figure 4b, the output characteristics (*I*_ds_–*V*_ds_ curves) of the SnS_2_ over h‐BN substrate are measured at various gate voltages (varying −60–60 V). The *I*_ds_–*V*_ds_ curves are recorded by sweeping the biasing voltages from −2 to +2 V and plotted in linear scale. The *I*_ds_–*V*_ds_ curve at each gate voltage demonstrates a linear relationship between the voltage and current values, indicating that the SnS_2_ FET being tested exhibits pure Ohmic behavior. The Ohmic behavior in SnS_2_ is due to the low work function of Ti metal (4.3 eV). This low work function of Ti metal causes a low Schottky barrier height at the metal‐semiconductor interface. This enables charge carriers to flow from the Ti metal into the SnS_2_ channel, resulting in linear *I*_ds_–*V*_ds_ curves with low resistance.^[^ ^55^, ^56^, ^57^, ^58^ ^]^

Moreover, SnS_2_ exhibits *n*‐type characteristics as explained earlier from the transfer curve, which means that it has an excess of negatively charged electrons. Therefore, when the gate voltage is increased from 60 to −60 V, the negative charges induce an electric field that modulates the fermi‐level within *n*‐type SnS_2_. At *V*_g_ <0 V, the fermi‐level shifted away from the conduction band that reduces the number of free electrons available for current flow in *n*‐type SnS_2_. As a result, the output current of the device decreases dramatically.

Furthermore, the SnS_2_ FET device was utilized as a biosensor to investigate its response against the PLCB construct and streptavidin, separately. Figure 4c displays a comparison of the transfer curves obtained before and after the introduction of the PLCB construct and streptavidin to the SnS_2_ FET surface while maintaining a constant drain‐source voltage (*V*_ds_ = 1 V). The π–π interaction between the SnS_2_ sheet surface and the attached PLCB molecules results in a significant increase in the current during positive gate voltages, along with a left shift in the threshold voltage.

The PLCB molecule retains high electron affinity due to the presence of functional groups with electron‐withdrawing properties, such as the carboxylate and phosphate groups in the biotin part of the molecule. These functional groups have a strong ability to pull electrons away from neighboring atoms or molecules, which can make the molecule more electron‐rich and lead to a stronger attraction to additional electrons. The high electron affinity of the PLCB molecule could be relevant to its interactions with other materials, as it may have a tendency to donate or accept electrons depending on the specific environment and conditions.

Subsequently, the SnS_2_ FET surface was covered with a solution of 2.5 µL, containing 1 pm streptavidin. The interaction between the PLCB and streptavidin molecules resulted in the sharing of charge, due to their strong non‐covalent bonding, causing a significant decrease in the output current and a right shift in the threshold voltage, as shown in Figure 4c. Moreover, Figure 4d presents the output curves that confirm the coupling of streptavidin with our engineered PLCB construct. The *I*_ds_–*V*_ds_ curves were recorded keeping the gate voltage constant at (*V*_g_ = 40 V) and compared before and after the introduction of the PLCB and streptavidin over the SnS_2_ device surface. The detection mechanism for streptavidin on the SnS_2_/h‐BN platform involves functionalizing the device by pyrene‐based supporter construct (PLCB) solution over the SnS_2_/h‐BN channel by a fair π‐electron distribution (16 in pyrene vs 4 in SnS_2_), which enables stable π–π stacking, as shown in Figure S5 (Supporting Information). Moreover, our chemically modified pyrene, coupled via Solid Phase Peptide Synthesis, allows charge transfer interactions with SnS_2_, involving electron transfer. Finally, the drop‐casting streptavidin solution enables robust non‐covalent interactions between streptavidin and biotin, coupled in our engineered PLCB. The strong interactions (non‐covalent), with dissociation constants in the order of ≈10^−14^ mol L^−1^ is responsible for sterpatavidin interaction with biotin.^[^ ^36^ ^]^

Further, when a drop of streptavidin solution was introduced, the output current significantly decreased, which can be attributed to charge sharing between the PLCB molecules and streptavidin because of their significant non‐covalent interactions. The combination of these multiple (hydrogen bonding and van der Waals forces) interactions that arise due to the charge interactions collectively contributes to the strength of the binding between the target molecule (streptavidin) and our PLCB construct (see Figure S2, Supporting Information).

**Figure 5:**
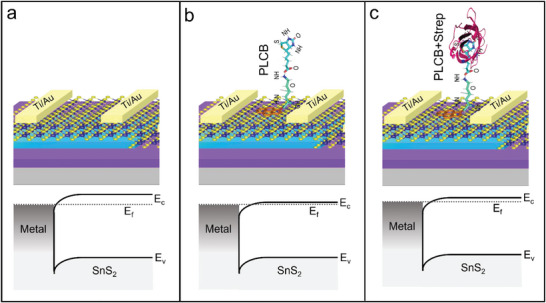
The energy band diagram of the SnS_2_ FET before and after functionalization with PLCB and streptavidin. The Fermi level *E*_f_ is denoted by the dotted line, while the conduction band and valence band are illustrated by *E*_c_ and *E*_v_. a) pristine SnS_2_ FET over the h‐BN b), SnS_2_ FET after functionalization with PLCB supporter construct c), and SnS_2_ FET after capturing the target analyte of streptavidin.

The variation in SnS_2_ device current after functionalization and introducing the streptavidin is interpreted with the energy band diagram. For the pristine SnS_2_ FET over the h‐BN substrate, the alignment of the fermi level (*E*_f_), valence band (*V*_b_), conduction band (*C*_b_), and barrier height (*Φ*_b_) among Ti/Au and SnS_2_ are presented in **Figure** **5**a. During the equilibrium condition, the fermi level exists near the conduction band but away from the valence band because of the *n*‐type nature of the SnS_2_ as it possesses the electrons as majority charge carriers.^[^ ^59^ ^]^ Once the SnS_2_ surface has been functionalized with the PLCB supporter construct, the barrier height is reduced because of the charge sharing from the PLCB supporter construct to the SnS_2_ surface. The SnS_2_ becomes increasingly electron‐rich and its fermi level moves closer to the conduction band as presented in Figure 5b. As the PLCB molecule retains high electron affinity due to the presence of functional groups in the biotin. These functional groups have a strong tendency to electrons, which can make the SnS_2_ surface more electron‐rich. Further, when the streptavidin is introduced to the SnS_2_ FET, the pre‐stacked PLCB molecules on its surface have the strong ability to capture the streptavidin because of its high electron affinity. The streptavidin protein has a tetrameric structure, with each subunit containing a binding pocket that is capable of accommodating even a single biotin molecule. As the streptavidin is captured by PLCB molecules via strong non‐covalent interactions, some of the charges are shared among them and the SnS_2_ fermi level shifted downward slightly as depicted in Figure 5c, resulting in a slight depression in current. Here, the barrier height increases slightly compared to the PLCB functionalized device, but it is still lower than the barrier height of the pristine device. As a result, the current increases compared to the pristine device but slightly decreases compared to the PLCB functionalized device.

### 2.4 Real‐Time Detection

**Figure 6:**
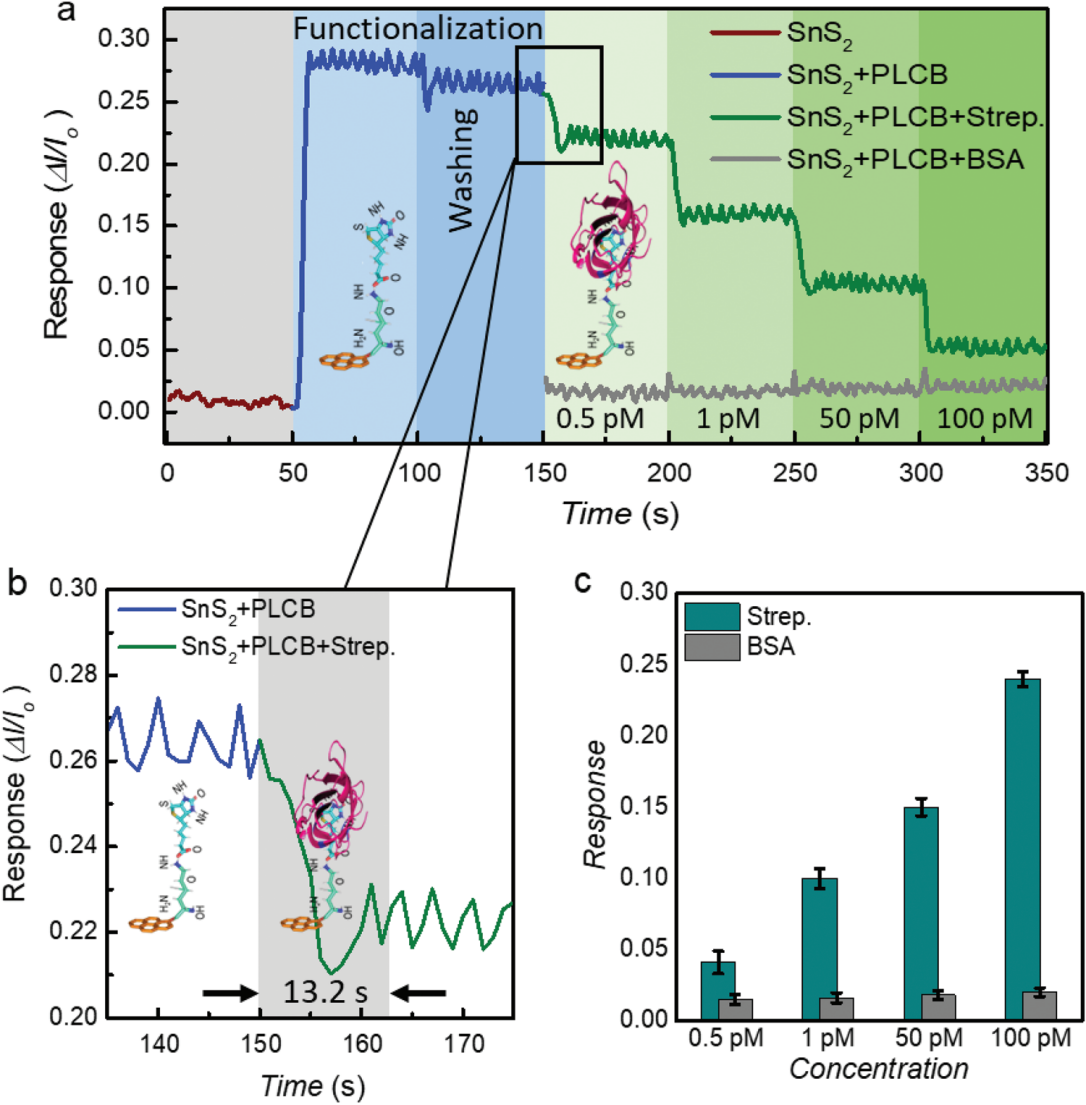
a) The real‐time response of the SnS_2_ FET over the h‐BN substrate against the various streptavidin concentrations, ranging from 0.5 to 100 pm. The device's current was monitored after each step over time. The Brown line represents the current of the pristine SnS_2_ FET over h‐BN. The Blue line shows the response of the device to the PLCB supporter construct, while the current against the streptavidin of various concentrations is plotted in green color. The Grey line depicts the current against the non‐targeted protein bovine serum albumin (BSA) protein. b) The fast time response of the SnS_2_ FET over the h‐BN substrate is recorded against the 0.5 pm concentration of streptavidin, and it was found to be equivalent to 13.2 s. c) The selectivity test of the SnS_2_ FET was performed with the BSA, a non‐targeted protein with a molecular weight equivalent to that of streptavidin. As shown in the grey bar graphs, the device did not exhibit any response to increasing concentrations of BSA (from 0.5 to 100 pm). All experimental investigations are repeated using three different devices (n = 3) for the elimination of possible variation in marked values. The data was plotted keeping> 95% of confidence interval (CI). The standard error of the mean (SEM) for all sets of measurements lies ≤ 0.002 along with the standard deviation (SD) ≤ 0.003 and variance (*P*) ≤ 0.001. While all the measurements with SEM close to zero, ± 2SD and *P* ≤ 0.01 are considered significant. Statistically, the lower detection limit (0.5 pm) of streptavidin can be considered reliable while the result is supported by the control sample, by mean ± 5 (SD).

For real‐time detection, we aimed to detect streptavidin, a commonly used protein in biotechnology and biosensing applications using a Keysight B1500‐A semiconductor parameter analyzer. The fixed drain‐source voltage (*V*_ds_ = 1 V) is applied to the channel material and the gate voltage is fixed at *V*_g_ = +40 V. Initially, the output current is recorded for the pristine SnS_2_ FET device until it is saturated. The normalized device response (Response = ΔI/I_o_ = (I‐I_o_)/I_o_) is estimated and plotted as a function of time as shown in **Figure** **6**a. Here I is the current detected while measurement while I_o_ is the initial baseline current. During the functionalization step, a small drop of phosphate‐buffered saline (PBS) solution containing 1 nm of PLCB supporter construct is dropped over the channel area using a micropipette. This device functionalization results in enhanced device sensitivity against the target analyte. After introducing the PLCB supporter construct to the surface of SnS_2_, a clear elevation in device output current is noted, which can be seen in Figure 6a (in blue color), indicating the successful functionalization of the SnS_2_ FET as a biosensor. Our self‐designed pyrene‐based supporter construct helps to control the alignment of the supporter molecule and its fine stacking onto the surface of SnS_2_ (see Figure S5, Supporting Information). Moreover, its small length ruled out the noise signal readouts and various effects such as Deby's screening effect.^[^ ^60^ ^]^

Finally, for the selective detection of the target analyte, a small droplet of PBS solution containing a 0.5 pm concentration of streptavidin is poured onto the device, meanwhile, the device output current is recorded (in green color) as depicted in Figure 6a. The output current and response time for SnS_2_ channel material are estimated and compared over both substrates of h‐BN and SiO_2_ as shown in Figure S6a–c (Supporting Information). The SnS_2_ FET has not responded against the 0.5 pm streptavidin concentration over the SiO_2_ substrate, but the same concentration is significantly detected over the h‐BN substrate. This could be attributed to the surface functionalization of the SnS_2_ FET over the SiO_2_ substrate that may not be optimal for streptavidin binding, leading to poor sensitivity as compared to the h‐BN substrate. Furthermore, the SnS_2_ FET's response is recorded over the h‐BN substrate against the various concentrations of the streptavidin varying from 0.5‐100 pm as indicated in Figure 6a. During the electrical measurement, the response current increases gradually by increasing the streptavidin concentration, but the response time decreases as the concentration is increased from 0.5 to 100 pm. The SnS_2_ FET exhibited a fast time response time of 13.2 s against the lowest streptavidin concentration of 0.5 pm as presented in Figure 6b. Furthermore, to test the selectivity of the device, we also investigated its response to bovine serum albumin (BSA), a non‐targeted protein having a molecular weight equivalent to streptavidin. We found that the device exhibited no significant response to any concentration of BSA compared to streptavidin as shown in Figure 6c, indicating that the device was selective toward streptavidin. Figure S7 (Supporting Information) also shows the results of the extended selectivity testing employing a variety of target analytes, including BSA, Lysozyme, and streptavidin. The detailed comparison of the SnS_2_ FET over the h‐BN substrate is presented in **Table** **1**. For the target analyte, the SnS_2_ FET as a biosensor device exhibited high sensitivity, prompt response, and detection limits less than the picomolar range.

**Table 1:** Comparison of the SnS_2_/h‐BN device performance with various types of biosensors.

Sensor Type	Materials	Target	Detected Concentration	Response Time	Reference
FET	MoS2	streptavidin	1 fm	23 min	[62]
vdW BJT	MoTe_2_/GeSe/MoTe_2_	streptavidin	5 pm	7.5 s	[36]
Anodic Alumina	ATPES	streptavidin	1 µg mL^−1^	10 min	[63]
FET	Graphene	Exosomes	0.1 µg mL^−1^	30 min	[64]
Metal‐mesh	Ni and Si	streptavidin	900 nm	2 min	[65]
FET	rGO	DNA‐Influenza	5 pm	2–4 h	[66]
Electro‐chemical	PdNPs/Si ITO	Dopamine	25 nm	–	[67]
FET	Si‐Nanowires	PSA	1 nm	8 min	[68]
FET	Sulpho‐NHS‐LC	streptavidin	3 mm	–	[69]
FET	WSe_2_	SARS‐CoV‐2 spike protein	25 fg µL^−1^	–	[70]
FET	Si‐nano wires	streptavidin	2 pm	80 min	[61]
Nanopore	peptide probes	protein	40 pm	15 min	[71]
FET	SnS_2_/h‐BN	streptavidin	0.5 pm	13.2 s	This work*

## 3 Conclusion

In this study, we successfully developed a biosensing device that utilizes high‐quality Tin sulfide and hexagonal boron nitride crystals for the accurate and rapid detection of streptavidin protein. For the streptavidin detection by SnS_2_ FETs, a supporter construct of biotin was coupled with a Pyrene conjugated Lysine to functionalize the surface of the biosensor device. This is because the biotin part of the molecule binds tightly to the streptavidin protein, while the Pyrene coupled with Lysine is stacked well over the hexagonal surface of SnS_2_ via π–π interaction. It allows the precise targeting of the streptavidin protein away from the sensing surface through this self‐designed supporter construct of PLCB. The SnS_2_ FET device functionalization and its response against the target analyte were characterized using Raman spectroscopy and electrical characterizations. During the real‐time measurements, the lowest streptavidin concentration of 0.5 pm was detected within 13.2 s without any time‐consuming functionalization. Additionally, the selectivity of the device is investigated by measuring its response to Cow‐like serum egg whites protein (BSA) with a molecular weight comparable to that of our target analyte (streptavidin). Although the use of h‐BN could make the fabrication process a little complex but it enhances the SnS_2_ device performance and improves its detection limit as compared to previously reported biosensors.^[^ ^36^, ^61^ ^]^ SnS_2_/h‐BN FET reduced the signal‐to‐noise ratio and promptly responded against the target analyte. Furthermore, the stability of SnS_2_ and h‐BN ensures prolonged sensor performance, while their minimized electronic interference reduces cross‐talk. These results demonstrate the high sensitivity and prompt response of the SnS_2_/h‐BN FET biosensor against the selective proteins, which could have significant applications in numerous fields including point‐of‐care diagnostics, agriculture, drug discovery, and environmental monitoring.

## 4 Experimental Section

### Device Fabrication

The high‐quality crystals of hexagonal boron nitride (h‐BN) and tin‐disulfide (SnS_2_) were purchased from a commercial supplier (2D semiconductor, N. Hayden Rd Suite 210–380 Scottsdale, AZ 85 251, USA) that specializes in 2D semiconductors. To prepare devices, a technique of mechanical exfoliation was utilized, in which a piece of Scotch tape was used to peel off thin sheets of the 2D semiconductor materials. This process allowed to produce ultra‐thin sheets of 2D materials up to a few layers thick and explore their intrinsic properties. To fabricate the SnS_2_/h‐BN FET devices on a *p*‐doped Si/SiO_2_ substrate, first, a few layers of thick h‐BN was transferred onto the substrate followed by the transfer of a few‐layer SnS_2_ over it. The SnS_2_ was transferred in such a way that half the part was over h‐BN and half of SnS_2_ was over the SiO_2_ substrate. Prior to the transfer of h‐BN and SnS_2_, the SiO_2_‐coated (*p*‐Si) substrate was cleaned using pure acetone followed by methanol, and deep‐UV light (DUV, Bachur & Associates, Santa Clara, Canada) to ensure that the surface was free from any impurities that could affect the quality of the experimental results. To pattern the electrodes, both photolithography and electron beam lithography techniques were used. Polymers EL‐9, SPR, and PMMA‐950 were spin‐coated onto the substrate loaded with SnS_2_/h‐BN van der Waals heterostructure to create big patterns for photolithography. For the microelectrodes, a scanning electron microscope (SEM, TESCAN, Ltd., Seoul, Korea) was used. Finally, for the electron beam lithography, the Quantum Alpha was utilized. The 5/70 nm thick Ti/Au metal electrodes were then deposited on the SnS_2_ flakes using thermal evaporators in a high‐vacuum (10^−6^ torr) metal deposition chamber.

### Materials Analysis

Atomic force microscopy (AFM, I‐Nexus Co., Ltd., Seoul, Korea) was used to analyze the topography and thickness of each 2D material, as it is a powerful tool that can provide information at the nanoscale level. The height profile graphs obtained from AFM analysis were used to estimate the thickness of SnS_2_ and h‐BN flakes, which were found to be ≈7.1 and 4.5 nm, respectively. The high‐resolution images obtained through AFM analysis allowed for detailed visualization of the surface topography of the materials, providing valuable information about their structure and properties.

Moreover, the Raman spectra for SnS_2_ and h‐BN were obtained using a Raman microscope (RamanScope III, Renishaw) at room temperature with a 532 nm laser and a 50x (and 100x) objective lens. The Raman spectra were recorded for pristine SnS_2_ and h‐BN devices, the devices functionalized with the pyrene‐based receptor PLCB, and the devices after capturing streptavidin.

### Electrical Characterizations and Real‐Time Detection

The electrical performance of the SnS_2_/h‐BN FET was evaluated using the Keysight B1500‐A semiconductor parameter analyzer coupled with a state‐of‐the‐art probe station. For electrical signal measurement, the biasing voltage and gate voltage were applied to the source and gate using Signatone probe tips model SE‐BC made of beryllium copper. The FET was connected to the parameter analyzer using the probe station, and a range of voltages was applied to the FET to measure the corresponding current. To estimate the device response to the streptavidin, the detected electrical response signal was normalized as [ΔI/I_o_] = (I−I_o_)/I_o_, where ΔI is the change in current from the initial current I_o_, and real‐time current I. The initial current I_o_ was measured before introducing the PLCB and target analyte. Moreover, all real‐time measurements were conducted in an ambient environment, to detect the streptavidin protein. For real‐time detection, the fixed *V*_ds_ = 1 V was employed in the FET device, and streptavidin of varying concentrations was introduced to the device surface using a micropipette of 0.1–2.5 µL range.

### Statistical Analysis

The pre‐processing and plotting of the experimental data was performed using Origin Pro 9.0 and GraphPad Prism 8.0 software. For each statistical analysis, the sample size (*n*) was fixed to 3. The spectra results of SnS_2_ channel material over various substrates, including SiO_2_ and h‐BN, at each stage of device operation (such as after functionalization and protein detection), were plotted and analyzed using Raman spectra analysis. All the peak properties (including resonance and defects) were calculated using Gaussian fit through multi‐peak fitting while keeping the coefficient of determination (R^2^)> 95%. The sensitivity of the substrate in the presence of h‐BN was analyzed by measuring and statistically analyzing the Raman spectra, as presented in Figure 3b–d. The standard error of the mean (SEM) was calculated and found to be ≤0.005, indicating promising results and consistency in measurement. The standard deviation (SD) was also calculated, and it appeared to be ≤0.09, while ±2SD was considered reliable. The variance (*P*) was calculated, and it was found to be close to zero (≤0.009), with significance defined as *P* ≤0.01. However, the statistical values for peak intensity ratio (*A*_1g_/*E*_g_) exceeded the abovementioned limit due to the ultra‐high sensitivity of the substrate. Therefore, the coefficient of variance (CV) was calculated, and it was found to be ≤9.0 for all measurements, with a CV between <30 considered acceptable. After confirming the sensitivity induction to the substrate in the presence of h‐BN, the device was utilized for selective protein (streptavidin) detection followed by its functionalization via the PLCB construct. The SEM values for all the FWHM‐*A*_1g_ peak measurements lie ≤0.09, and the values approached zero, confirming reliable measurement, as shown in Figure 3d. Similarly, the SD was found to be ≤0.17, with ±2SD being considered reliable. The variance (*P*) was ≤0.029, with significance defined as *P*≤0.01. The statistical values for peak intensity ratio (*A*_1g_/*E*_g_) somehow exceeded the abovementioned limit due to ultra‐high substrate sensitivity. However, the coefficient of variance (CV) was again found to be ≤9.80 for all sets of measurements. Finally, the device was subjected to electrical measurements, and the current response was noted utilizing three separate devices (*n* = 3) for error elimination. The average data was plotted (keeping a confidence interval >95%), as shown in Figure 6a–c. The SEM values for all the measurements were ≤ 0.002, and the SD was found to be ≤ 0.003, with the SEM near zero and ±2SD being considered reliable. The variance (*P*) was close to zero, with the coefficient of variance (CV) lying in the range of 1.25–8.54 for all experimental measurements. Regarding the streptavidin detection, 0.5 pm was considered a reliable concentration after comparing it with the control measurements by mean ± 5 (SD). In general, the significance was marked as *P* ≤0.01. Minitab‐17 statistical software was used for all of the statistical analyses.

## Conflict of Interest

The authors declare no conflict of interest.

## Author Contributions

This project was designed by S.N., G.D., and D.K. G.D. and S.N. fabricated the devices and characterized the materials. Z.M.S. designed and synthesized the PLCB support construct. G.D., S.N., and Z.M. S. extracted the data. G.D., D.K., Z.M.S., I.R., and J.E. analyzed the data. M.S.A., B. B., and A. R. helped to provide the resources. G.D. and D.K. supervised the project. And G.D. and S.N. wrote the final manuscript.

## Supporting information

Supporting Information

## Data Availability

The data that support the findings of this study are available from the corresponding author upon reasonable request.
