# Supplementary material for: A Novel Biosensing Approach: Improving SnS2 FET Sensitivity with a Tailored Supporter Molecule and Custom Substrate
Source: Adv Sci (Weinh). 2023 Oct 20;10(33):2303654. doi: 10.1002/advs.202303654 (PMC10667857; doi:10.1002/advs.202303654)
Supplement: Supplementary file 1 — Supporting Information [file ADVS-10-2303654-s001.pdf]

ADVANCED SCIENCE
Open Access

Supporting Information

for *Adv. Sci.*, DOI 10.1002/advs.202303654

A Novel Biosensing Approach: Improving SnS_2 FET Sensitivity with a Tailored Supporter Molecule and Custom Substrate

Sobia Nisar, Beriham Basha, Ghulam Dastgeer, Zafar M. Shahzad, Honggyun Kim, Iqra Rabani, Aamir Rasheed, M. S. Al-Buriahi, Ahmad Irfan, Jonghwa Eom and Deok-kee Kim**

Supporting Information

A Novel Biosensing Approach: Improving SnS_2 FET Sensitivity with a Tailored Supporter Molecule and Custom Substrate

Sobia Nisar[1], Beriham Basha[2], Ghulam Dastgeer *[3], Zafar M. Shahzad[4], Honggyun Kim[5], Iqra Rabani[6], Aamir Rasheed[7], M. S. Al-Buriahi[8], Ahmad Irfan[9], Jonghwa Eom[3], Deok-kee Kim*[1,5]

[1]Department of Electrical Engineering and Convergence Engineering for Intelligent Drone, Sejong University, Seoul 05006, Republic of Korea.

[2]Department of Physics, College of Sciences, Princess Nourah Bint Abdulrahman University, P. O Box 84428, Riyadh 11671, Saudi Arabia.

[3]Department of Physics and Astronomy, Sejong University, Seoul 05006, Republic of Korea.

[4]SKKU Advanced Institute of Nanotechnology (SAINT) and Department of Chemical and Polymer Engineering, Sungkyunkwan University, Suwon, 16419, Republic of Korea.

[5]Department of Semiconductor Systems Engineering, Sejong University, Seoul 05006, Republic of Korea.

[6]Department of Nanotechnology and advanced materials engineering, Sejong University, Seoul 05006, Republic of Korea.

[7]Department of Chemistry, University of Ulsan, Ulsan, 44610, Republic of Korea

[8]Department of Physics, Sakarya University, Sakarya, Turkey.

[9]Department of Chemistry, College of Science, King Khalid University, P.O. Box 9004, Abha 61413, Saudi Arabia.

Corresponding Authors: Ghulam Dastgeer and Deok-Kee Kim

Email: gdastgeer@sejong.ac.kr and deokkeekim@sejong.ac.kr

Detailed reaction mechanism for the synthesis of PLCB

The pyrene-lysine-biotin construct, a molecule used to support PLB, was created using Fmoc chemistry. The H-Rink amide resin (substitution value = 0.54 mmol/g) was purchased from PCAS BioMatrix company. A benchtop manually operated solid-phase peptide synthesizer (SPPS) was used for the synthesis process. Before starting synthesis, a fixed quantity of resin was swelled using DMF for half an hour. For deprotection using 20% piperidine in N, N-dimethylformamide (DMF), a standard set of conditions was used, i.e., heating at 90 °C followed by 75 °C for 15 s and 50 s respectively. The resin was washed with DMF and dichloromethane (DCM) between the deprotection and coupling steps. The synthesis began by coupling Fmoc-Lys (Biotin)-OH moiety in assistance with activated amino acid (DIPEA). The coupling reaction was performed by adding 0.5 M diisopropyl carbodiimide (DIC) and 1 M Oxyma in the SPPS synthesizer. The reaction was performed at the same temperature conditions mentioned above but increased reaction time by 55%. After deprotection, 0.2 M 1-pyrene butyric acid solution was used for coupling keeping the same concentration. The final product was dried and cleaved from resin support using trifluoroacetic acid (95%), tri-isopropyl silane (2.5%), and the rest deionized water. The product was filtered under a nitrogen atmosphere, precipitated using ether (diethyl ether), and lyophilized to prepare a solution of desired concentration for all experimental investigations. The concentration of our PLCB was estimated by measuring the absorbance of its solution at 335 nm. An optimized concentration of 1 nM was used for substrate functionalization to avoid stacking. For further details on the reaction mechanism, please refer to **Figure S1a**. The synthesized PLCB was characterized via UV-spectra for the presence of pyrene and successful biotinylation, as shown in **Figure S1b**. The appearance of a clear peak at ~ 202 nm confirms biotinylation, whereas, a clear and sharp peak at 335 nm confirms the conjugation of pyrene. At each stage of synthesis, the Kaiser test was used to confirm coupling and deprotection as shown in **Figure S1c.**

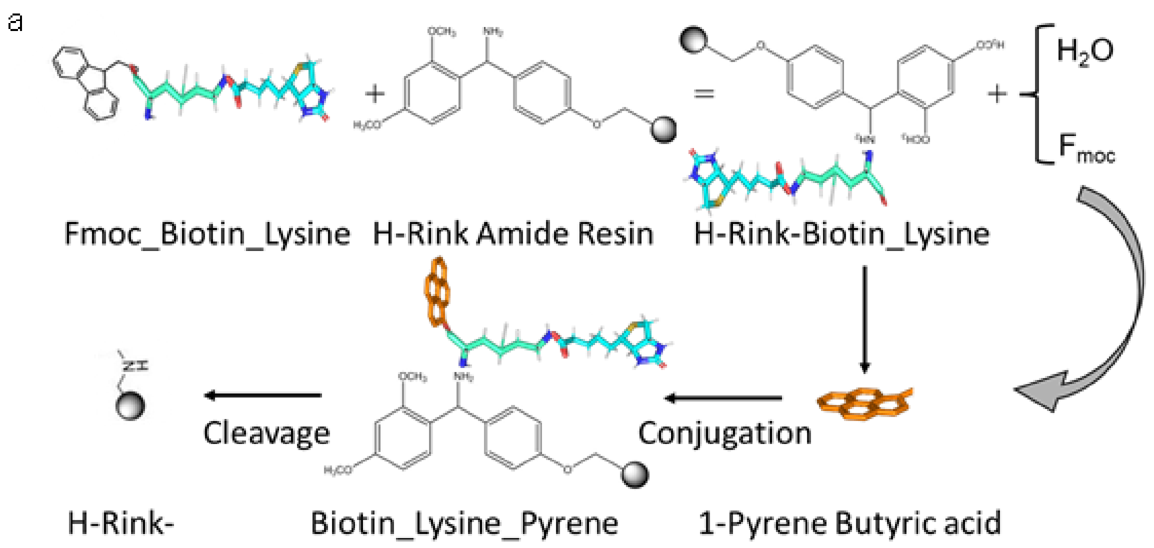
a
H-Rink Amide Resin
H-Rink-Biotin_Lysine
Fmoc_Biotin_Lysine
Cleavage
Conjugation
H-Rink-
Biotin_Lysine_Pyrene
1-Pyrene Butyric acid

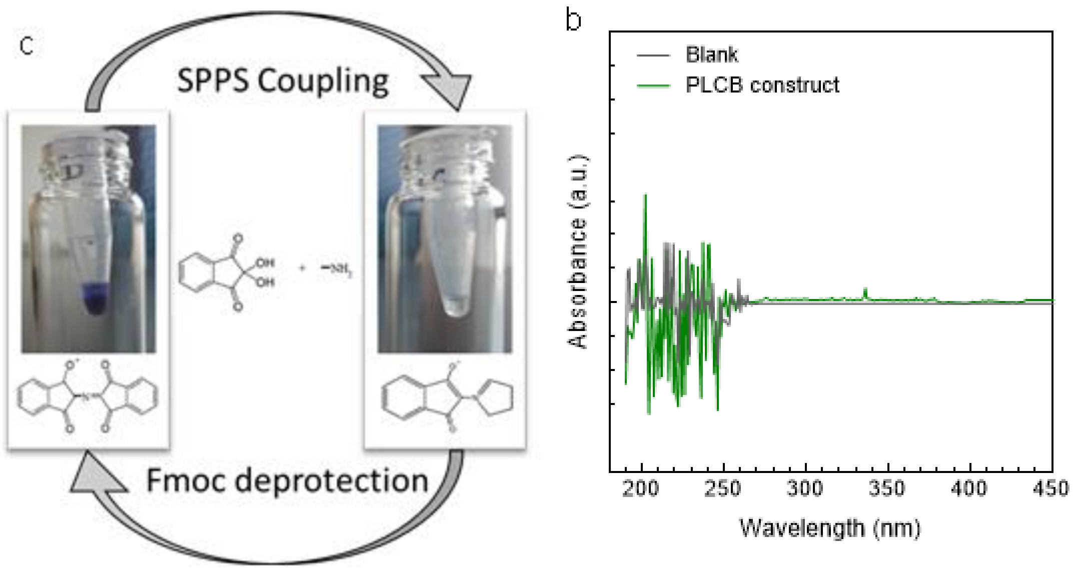
c
SPPS Coupling
Fmoc deprotection
b
Blank
PLCB construct
Absorbance (a.u.)
Wavelength (nm)
200
250
300
350
400
450

Figure S1. (a) The synthesis and characterization of our engineered PLCB construct. **(a)** The reaction mechanism in which the de-protected H-Rink amide resin is coupled with Fmoc-protected Biotin-Lysine. Further, the coupling reaction of 1-purene butyric acid via standard coupling conditions and the final cleavage to get the desired product. For coupling, a standard solution containing DIC and Oxyma was used. For de-protection, 20% piperidine in DMF was used. **(b)** The middle-long wave UV spectra were used to characterize the successful synthesis of the PLCB construct. More specifically, the presence of biotin (sharp peak at 202 nm) is confirmed along with the pyrene conjugation (sharp peak at 335 nm) confirmation. **(c)** In all synthesis reactions, the coupling (colorless beads) and de-protection (violet color beads) were confirmed by the Kaiser test.

PLCB and streaptavidin nteraction.

The target protein (streptavidin) has a strong interaction capacity (dissociation constant in the order of ~ 10^{-14} mol L^{-1}) for the biotin (coupled with the PLCB construct, facing upwards). The interaction is marked by a formidable non-covalent bond (illustrated in **Figure S2**), whereby charges on the PLCB construct were shared, actively participating in the binding process with the target molecule (streptavidin). There is a total of 10 amino acids binding with the biotin including eight polar groups binding with hydrogen bonds and two non-polar groups binding with the van der Waals forces[1]. This combination of forces is not applicable to any

other moiety present in the system, hence, making possible the precise and prompt capturing of streptavidin by the PLCB construct.

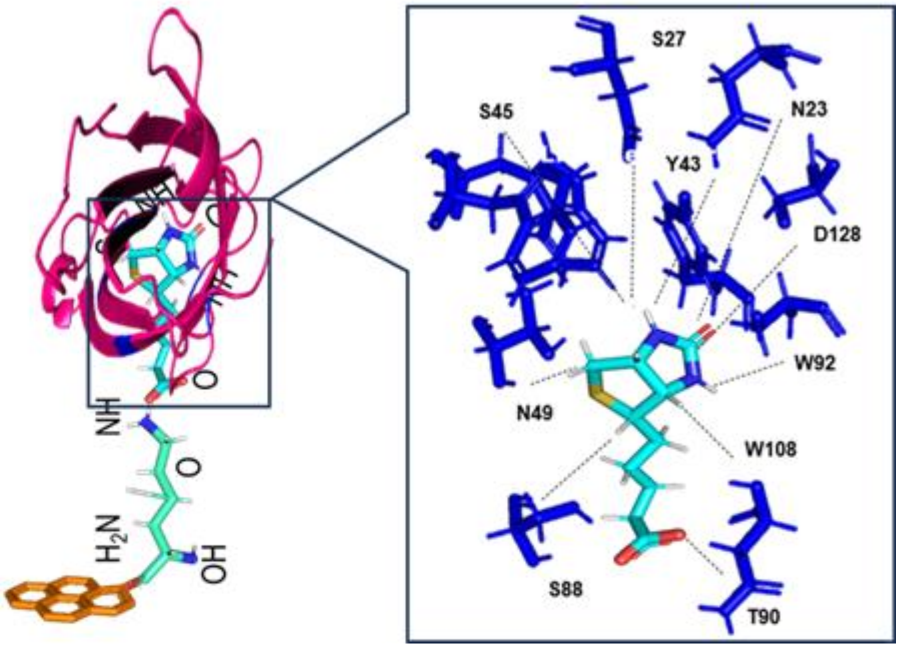

Figure S2: The binding of streptavidin by the biotin coupled onto the PLCB construct. The biotin is confined in the active site of streptavidin by ten different binding moieties involving eight hydrogen bonds (among polar groups) and two van der Waals interactions (among non-polar groups). The distances are estimated using the Pymol software and mentioned in Å. Insight represents a clear view of 10 binding sites per streptavidin molecule. The construct (PLCB) is engineered in such a way that upon functionalizing, the Pyrene moiety plays its role and aligns with the hexagonal surface of SnS_2. While, after functionalization, the biotin (facing upwards) is fully available to interact specifically with the target protein (streptavidin) with-which it has strong binding ability.

Raman Analysis of a few layers thick h-BN and SnS_2

The Raman spectra of the pristine h-BN exhibit an intense peak at ~1373 cm^{-1} which represents the E_{2g} peak, i.e., the high-frequency vibration mode of h-BN [2], as shown in **Figure S3a**. In the comparison of this peak with the bulk mode (E_{2g} located at 1366 cm^{-1}), a 6 cm^{-1} upshift can be observed which is inconsistent with the results reported earlier. Moreover, the FWHM of

the A_{1g} peak (main resonance) is related to the h-BN crystal dimensions which are about 10.17 cm^{-1} to suggest a few layers of h-BN with excellent crystallinity as compared to the results reported earlier[3].

The Raman spectra of pristine SnS_2 represent a clear and sharp peak around ~315.4 cm^{-1} (A_{1g}) and ~200 cm^{-1} (E_g) as shown in **Figure S3b**, confirming the pristine material nature. The appearance of two sharp peaks represents two active phonon modes of phase (2H) SnS_2 [4]. Moreover, the presence of a single peak in the 190-225 cm^{-1} range clearly indicates the pure crystalline SnS_2 at its ground state with 2H polytype. Applying the Gaussian fit, the full width at half maximum (FWHM) of A_{1g} (main resonance peak) is calculated, and it is 9.85, representing the crystalline nature of the material. Further, a sharp second resonance peak at 200 cm^{-1} represents the small number of layers of SnS_2 material as a broad peak at this position is attributed to the overlapping of a large number of layers[5]. Due to the pristine nature of SnS_2 material, the intensity ratio (A_{1g} / E_g) is very high and equivalent to 18. Moreover, the detailed Raman analysis of the SnS_2 surface before and after functionalization with PLCB and streptavidin is also illustrated in **Figure S3 c-d.**

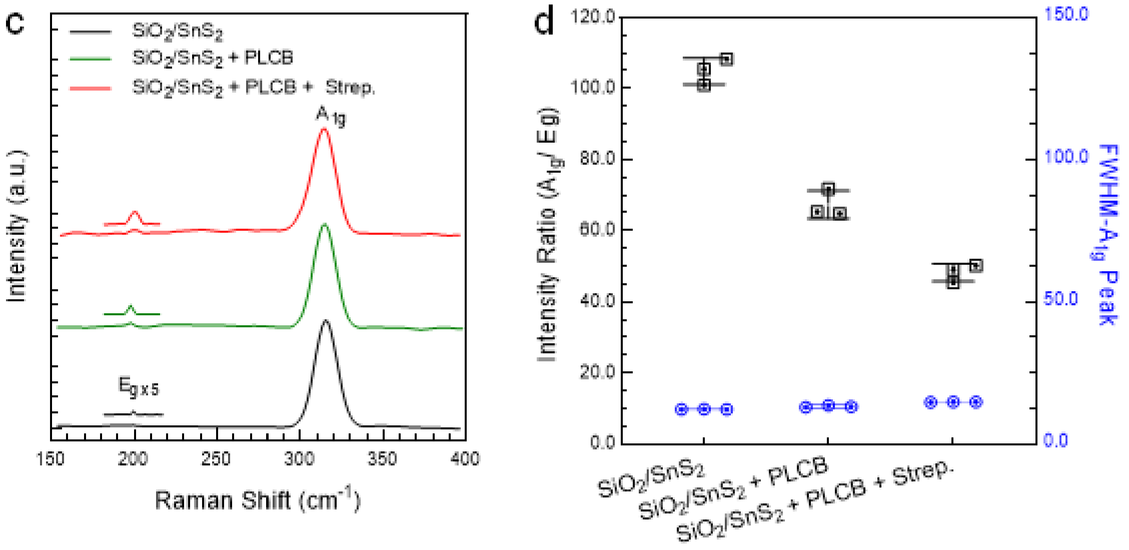
c
SiO2/SnS2
SiO2/SnS2 + PLCB
SiO2/SnS2 + PLCB + Strep.
A1g
Eg x 5
Intensity (a.u.)
Raman Shift (cm-1)
150 200 250 300 350 400
d
Intensity Ratio (A1g/ Eg)
FWHM-A1g Peak
0.0 20.0 40.0 60.0 80.0 100.0 120.0
0.0 50.0 100.0 150.0
SiO2/SnS2
SiO2/SnS2 + PLCB
SiO2/SnS2 + PLCB + Strep.

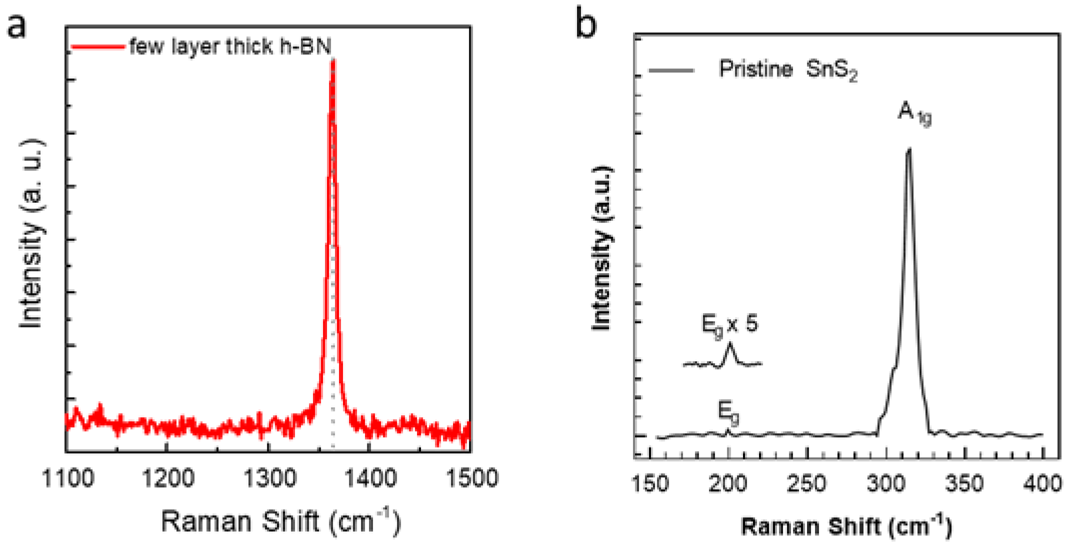
a
few layer thick h-BN
Intensity (a. u.)
Raman Shift (cm-1)
1100 1200 1300 1400 1500
b
Pristine SnS2
A1g
Eg x 5
Eg
Intensity (a.u.)
Raman Shift (cm-1)
150 200 250 300 350 400

Figure S3. (a) The Raman spectroscopy analysis of a few layers thick hexagonal boron nitride (h-BN) sample, shows characteristic Raman peaks at 1366 cm^{-1} (E_{2g} mode), confirming the crystalline quality of the h-BN material. **(b)** The Raman spectra of SnS_2 reveal the presence of A_{1g}, and E_{1g} in-plane modes of vibration, providing information on the vibrational and fine structural properties of the SnS_2 material. **(c)** The Raman analysis of the SnS_2 over the SiO_2 substrate after functionalization with PLCB supporter construct and streptavidin detection. **(d)** The intensity ratio and FWHM are plotted over the SiO_2 substrate for pristine SnS_2, after functionalization with PLCB, and after streptavidin detection.

Electrical characterizations of the SnS_2 over the Si/SiO_2 substrate.

The electrical characterizations of the SnS_2 channel materials were also investigated over the SiO_2 substrate as a comparative analysis. The red circle area is the SnS_2 FET device over the SiO2 substrate without h-BN as illustrated in **Figure S4a**. The transfer curves extracted at various biasing voltages are illustrated in **Figure S4b**, showing an ascending current as the biasing voltage is increased from 0.5-2.0 V. Even the SnS_2 FET exhibited a slightly high current over the SiO_2 substrate as compared to the h-Bn substrate as a result of the single-layer dielectric of SiO_2. Furthermore, the linear I_{ds} versus V_{ds} curves at various gate voltages are also plotted for the SnS_2 FET over the SiO_2 substrate, as shown in **Figure S4c**. The straight lines of I_{ds}-V_{ds} are presenting good Ohmic electrodes due to the negligible barrier height as shown in the energy band diagram of the main manuscript (Figure 5a-c).

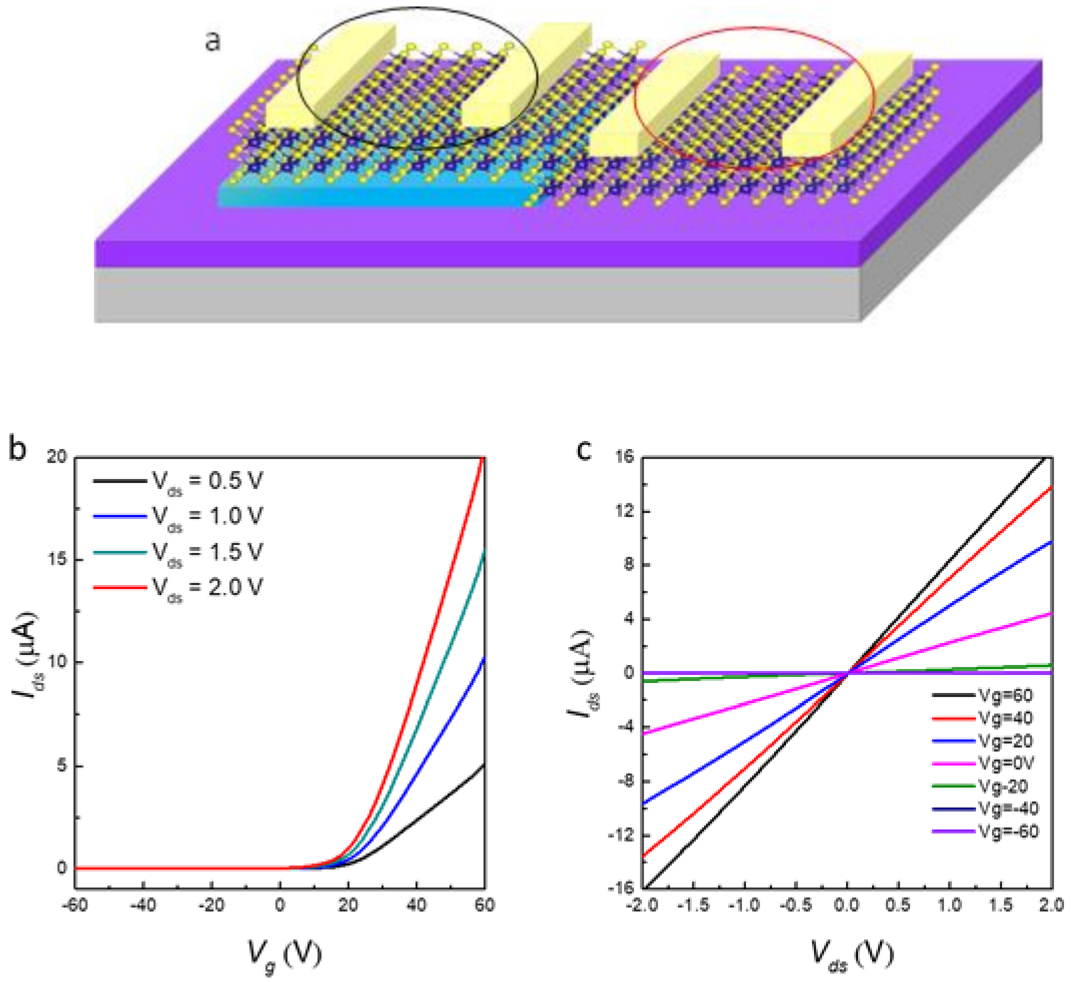

Figure S4: (a) The schematic diagram is illustrating the SnS_2 FET over the h-BN and SiO_2 substrates. The red circled area is showing the SnS_2 over the SiO_2 substrate while the black circled area is illustrating the SnS_2 over the h-BN substrate. **(b)** The transfer curves acquired from the SnS_2 FET over the SiO_2 substrate at various biasing voltages ranging from 0.5 to 2 V. **(b-c)** The output curves acquired from the SnS_2 FET over the SiO_2 substrate at various gate voltages changing from -60 to 60 V.

Stacking of PLCB over SnS_2 surface.

For the device functionalization, a solution (2.5 µL, 1 nM) containing our engineered pyrene-based supporter construct (PLCB) is poured over the channel containing SnS_2/h-BN. The hexagonal surface of the SnS_2 flakes provides an effective platform for the hexagonal pyrene rings (present in our PLCB construct) to assemble over it via π–π bonding without lying over as shown in **Figure S5**.

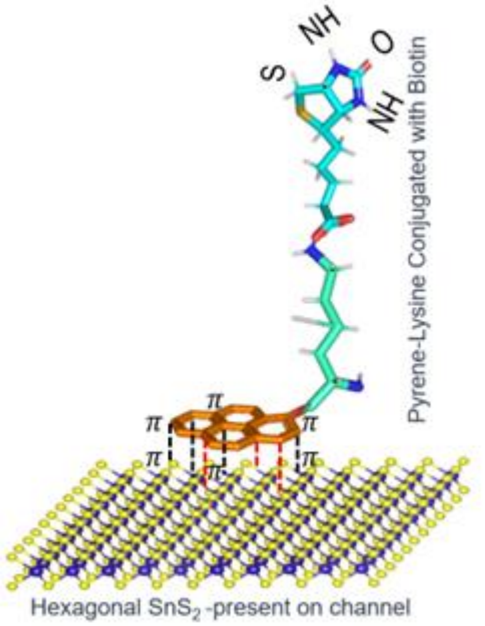

Figure S5: The SnS_2 (channel top layer) FET is functionalized with the PLCB construct. The Pyrene present in PCLB is stacked over the hexagonal surface of the SnS_2 sheet via π–π bonding (represented by Black lines). The transferring of electrons (those involved in loan pairs but not involved in bonding) from Pyrene-based PLCB to SnS_2 is presented in RED lines.

Pyrene is a polycyclic aromatic hydrocarbon consisting of four fused benzene rings which contribute a total of 16 π-electrons to its aromatic system. The aromaticity of pyrene arises from the presence of a continuous ring of π-electrons. Principally, pyrene can interact with other materials through various interactions, such as van der Waals forces, π-π stacking, and hydrogen bonding, etc. These interactions can occur between the aromatic rings of pyrene and other aromatic or electron-deficient systems in other materials (SnS_2), where the π-electron clouds of the aromatic rings align and interact through various attractive forces.

Real-Time Detection of Streptavidin via SnS_2 FET over h-BN and SiO_2 substrates.

At a fixed biasing voltage (V_{ds} = 1 V), the comparison of the SnS_2 FET over h-BN and SiO_2 substrates is plotted together, as shown in **Fig. S6a**. The real-time detection of the streptavidin over the h-BN substrate is showing more promising results as it detected the minimum concentration up to 0.5 pM while the SnS_2 FET over the SiO_2 substrate can detect the lowest concentration of streptavidin up to 1 pM. Furthermore, the response time of the SnS_2 device

over the h-B substrate is significantly lower than the SiO_2 substrate against the 1 pM streptavidin concentration as shown in **Fig. S6b**. Additionally, the selectivity test for the SnS_2 FET against streptavidin and BSA protein is also investigated at both substrates as shown in **Fig. S6c.**

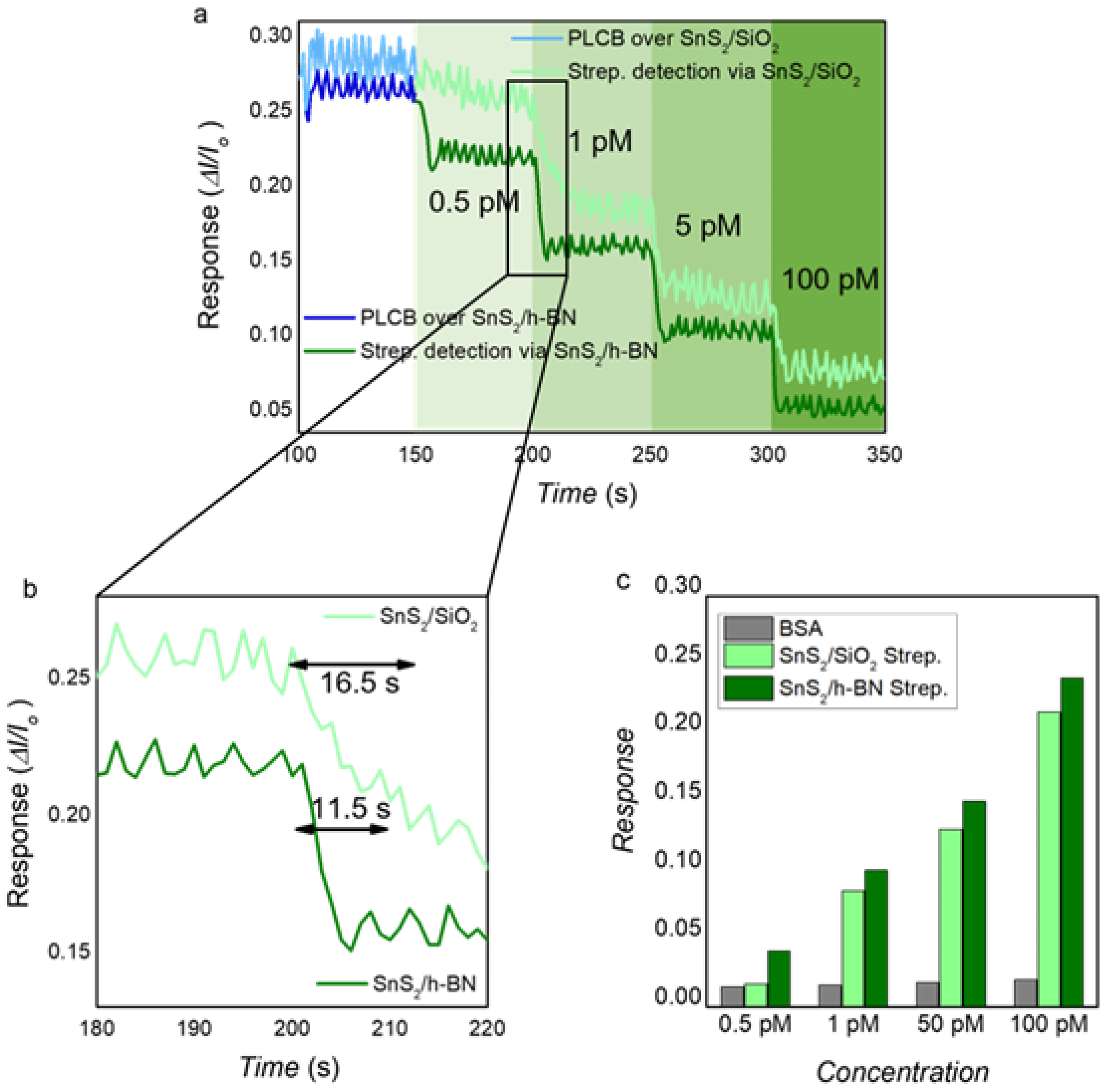

Fig. S6. (a) The real-time detection of streptavidin via SnS_2 FET over h-BN and SiO_2 substrates. At a fixed V_{ds} = 1 V, the response was recorded against the various concentrations of streptavidin at h-BN (in dark green color) and SiO_2 (in light green color) substrates. **(b)** The comparison of the response time of SnS_2 FET over h-BN and SiO_2 substrates is plotted together for in-depth evaluation. **(c)** The selectivity test of SnS_2 FET against the streptavidin and BSA

proteins plotted together. The response of the SnS_2 FET against the lowest concentration of 0.5 pM over the SiO_2 substrate is equivalent to the device response for BSA.

The SnS_2 FET exhibited faster and more sensitive detection of streptavidin over the h-BN substrate as compared to the simple SiO_2 substrate. This performance improvement may be due to several factors. Firstly, the h-BN substrate possesses a higher surface area and greater hydrophobicity than the SiO_2 substrate, which may facilitate the binding of streptavidin to the SnS_2 FET and enhance the detection sensitivity[6]. Secondly, the surface charge of the substrate could also play a role in the detection efficiency, with the surface charge of h-BN potentially promoting greater binding of streptavidin and therefore higher detection sensitivity. Thirdly, the properties of the SnS_2 FET itself may differ depending on the substrate, such as higher density or improved crystallinity when deposited on h-BN, contributing to the enhanced detection efficiency observed[7].

Selectivity Test.

Hence, we extended the selectivity testing by making various real simulated solutions of BSA (nearly similar M. Wt. of 66.4 kDa) and Lysozyme (the egg protein, having a different M. Wt. of 14.6 kDa) than our target protein (streptavidin). Each solution has the same analyte concentration of 0.5 pM. The results showed the selectivity of our device in capturing the target analyte (streptavidin) only as shown in **Figure S7**.

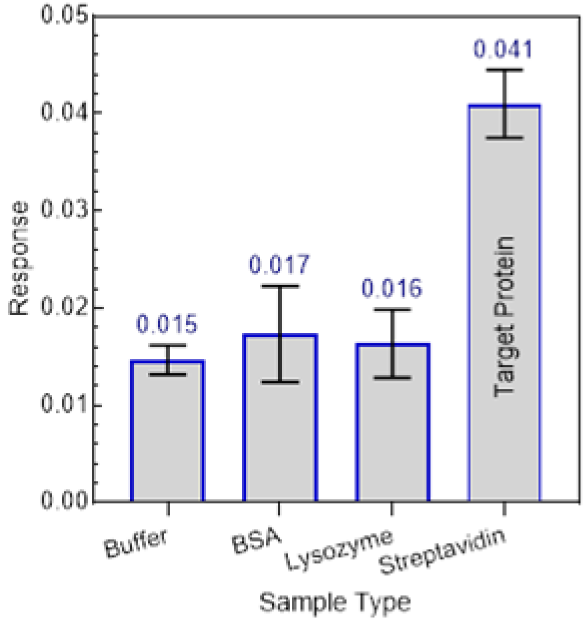

Figure S7: The device selectivity. The current response of the device is recorded for 0.5 pM of target protein (streptavidin) and non-targeted biomolecules of similar, Bovine Serum Albumin (BSA), and different (Lysozyme) molecular weight proteins. The results of selectivity analysis are triplicate using three different devices. Statistically, the standard error of the mean (SEM) is 0.0008 ≤ 0.002, the variance (P) lies ≤ 0.0001 while the SD lies in the range of 0.001-0.005 and the IQR also lies ≤ 0.009.

References

[1] F. Liu, J. Z. H. Zhang, Y. Mei, Scientific Reports 2016, 6, 27190.
[2] R. Geick, C. Perry, G. Rupprecht, Physical Review 1966, 146, 543; R. Arenal, A. Ferrari, S. Reich, L. Wirtz, J.-Y. Mevellec, S. Lefrant, A. Rubio, A. Loiseau, Nano letters 2006, 6, 1812.
[3] J. Yu, L. Qin, Y. Hao, S. Kuang, X. Bai, Y.-M. Chong, W. Zhang, E. Wang, ACS nano 2010, 4, 414.
[4] L. A. Burton, T. J. Whittles, D. Hesp, W. M. Linhart, J. M. Skelton, B. Hou, R. F. Webster, G. O'Dowd, C. Reece, D. Cherns, Journal of Materials Chemistry A 2016, 4, 1312; V. Hadjiev, D. De, H. Peng, J. Manongdo, A. Guloy, Physical Review B 2013, 87, 104302.
[5] A. Smith, P. Meek, W. Liang, Journal of Physics C: Solid State Physics 1977, 10, 1321.
[6] D. Horinek, A. Serr, M. Geisler, T. Pirzer, U. Slotta, S. Q. Lud, J. A. Garrido, T. Scheibel, T. Hugel, R. R. Netz, Proceedings of the National Academy of Sciences 2008, 105, 2842; G. Raffaini, F. Ganazzoli, Langmuir 2010, 26, 5679.
[7] N. Rohaizad, C. C. Mayorga-Martinez, M. Fojtů, N. M. Latiff, M. Pumera, Chemical Society Reviews 2021, 50, 619.
